# Generational Strengths Help Bridge Political Divides: An Analysis of Cross-Partisan Conversations

**DOI:** 10.1093/geroni/igaf122.3597

**Published:** 2025-12-31

**Authors:** Mary Rose Bell, Anna Pot, Laura Carstensen

**Affiliations:** Stanford University, Palo Alto, California, United States; Stanford University, Stanford, California, United States; Stanford University, Stanford, California, United States

## Abstract

Older adults’ socioemotional strengths, such as emotional stability and positivity, could be a valuable resource for navigating political discussions. When combined with younger adults’ curiosity and open-mindedness, these intergenerational strengths have the potential to foster respectful dialogue across political divides. We analyzed conversations from StoryCorps’ One Small Step initiative, which pairs individuals with different political views for 50-minute conversations. The sample included 105 conversations with young-young, old-old, and young-old dyads (N = 210 participants; M_young = 29 years, M_old = 71 years). Transcripts were analyzed using LIWC to measure the frequency of prosocial, polite, tentative, and positive words. Results showed that intergenerational (young-old) dyads used more polite words than young-young (b = 0.144, p < 0.001) and old-old dyads (b = 0.090, p = 0.033). Intergenerational dyads also used more prosocial words than young-young dyads (b = 0.146, p = 0.006). Additionally, intergenerational and old-old dyads used more positive words than young-young dyads, while intergenerational and young-young dyads used more tentative words (e.g., maybe, perhaps, wonder) than old-old dyads. These findings suggest that intergenerational dyads foster more positive and courteous political dialogue than same-age dyads. Social norms of deference may explain intergenerational dyads’ greater polite and prosocial word use. Moreover, the combined strengths of each generation (e.g., older adults’ positive reframing and younger adults’ open-mindedness) may underlie intergenerational dyads’ greater positive and tentative word use. In a time of marked political polarization in the U.S., intergenerational political discussions may offer a promising path to bridge divisions.

